# Visual Outcomes of Cataract Surgery in Patients with Previous History of Implantable Collamer Lens

**DOI:** 10.3390/jcm13154292

**Published:** 2024-07-23

**Authors:** Norma E. Del Risco, Chad L. Talbot, Kayvon A. Moin, Garrett N. Manion, Alex H Brown, Stephen M. Walker, Ping-Shou Zhong, Hanting Zhang, Phillip C. Hoopes, Majid Moshirfar

**Affiliations:** 1 Department of Ophthalmology, College of Medicine, University of Illinois, Chicago, IL 60612, USA; delrisc2@uic.edu; 2 Department of Ophthalmology, College of Osteopathic Medicine, Rocky Vista University, Ivins, UT 84738, USA; chad.talbot@ut.rvu.edu (C.L.T.); stephen.walker@ut.rvu.edu (S.M.W.); 3 Hoopes Vision Research Center, Hoopes Vision, 11820 S. State St., Ste. 200, Draper, UT 84020, USA; kmoin@hoopesvision.com (K.A.M.); pch@hoopesvision.com (P.C.H.); 4 Department of Ophthalmology, School of Medicine, Creighton University, Omaha, NE 68178, USA; garrettmanion@creighton.edu; 5 Department of Ophthalmology, College of Medicine, University of Arizona, Phoenix, AZ 85004, USA; alexhbrown@arizona.edu; 6 Department of Mathematics, Statistics, and Computer Science, University of Illinois, Chicago, IL 60612, USA; pszhong@uic.edu (P.-S.Z.); hzhan59@uic.edu (H.Z.); 7 John A. Moran Eye Center, School of Medicine, University of Utah, Salt Lake City, UT 84132, USA; 8 Utah Lions Eye Bank, Murray, UT 84107, USA

**Keywords:** refractive surgery, ICL, cataract surgery, myopia, spherical equivalent, astigmatism, visual outcomes

## Abstract

**Background/Objectives**: This retrospective case series analyzed visual outcomes in patients with a prior history of implantable collamer lens (ICL) implantation who underwent cataract extraction (CE). A secondary aim was to investigate the relationship between vault height and the rate of cataract development. **Methods**: Visual acuity and refraction measurements were collected after CE at one week, one month and six months. Vault height measurements were correlated to the time until symptomatic cataracts were removed. **Results**: A total of 44 eyes were analyzed at six months after CE with efficacy and safety indexes of 1.20 ± 1.11 and 1.50 ± 1.06, respectively. In addition, 70% of eyes had a post-operative uncorrected distance visual acuity (UDVA) within one line of pre-operative corrected distance visual acuity (CDVA). Refractive predictability at six months demonstrated that 43% and 69% of eyes were within ±0.25 D and ±0.50 D of SEQ target, respectively. Astigmatism measured by refractive cylinder was ≤0.25 D in 17% and ≤0.50 D in 34% of eyes pre-operatively compared to 40% and 60% of eyes, respectively, at six months post-operatively. Vault heights one week after ICL (*p* < 0.0081) and one week before CE (*p* < 0.0154) demonstrated a positive linear regression with the time until CE. **Conclusions**: This sample population achieved favorable visual outcomes six months after CE, similar to six months after ICL implantation. Patients with a history of ICL implantation will similarly have a good visual prognosis after CE.

## 1. Introduction

Amid the global myopia epidemic over the past few decades, largely driven by environmental factors, such as intense educational pressure, excessive use of electronic devices, and reduced outdoor time, the rates of myopia and high myopia have risen [1,2]. Consequently, the demand for refractive surgery has increased. The Implantable Collamer lens (ICL) is a type of posterior chamber phakic intraocular lens (pIOL) that offers a wide range of refractive correction for myopic patients (−3.00 to −20.00 D). When compared to corneal refractive surgeries, the advantages of Implantable Collamer Lenses (ICL) include the near-complete preservation of corneal biomechanics with a virtually zero risk of corneal ectasia [3], while maintaining a rapid visual recovery [4,5]. Its safety, efficacy, stability, and predictability make it a great option for those who would not qualify for other refractive procedures [6,7].

Despite being an excellent option for those who do not qualify for other refractive procedures due to its safety, efficacy, stability, and predictability, it is important to consider the potential side effects of ICL implantation [6,7]. The most commonly reported complication has been the development of cataracts with an incidence of 12.5% within 10 years of implantation in moderate to high myopes [7,8]. This risk, however, has been associated with early models of ICL and has significantly decreased with the introduction of the 360-micron central port model (Visian ICL V4c or EVO model). This model avoids the need for pre-operative or post-operative iridotomy and improves aqueous humor dynamics in the narrow space between the posterior surface of the ICL and the anterior surface of the crystalline lens [9,10].

Several long-term follow-up studies with the EVO pIOL have shown near-zero cataract rates, even in cases with a low vault [10,11,12], in contrast with secondary cataract risk observed in the previous generations of ICL [13]. A key factor contributing to ICL-induced cataracts in early models was pIOL vault height [14]. Low vaulting (<250 μm) in models without a central port is believed to hinder the circulation of aqueous humor in front of the crystalline lens, increasing the risk of metabolic cataract formation [15]. Therefore, successful ICL outcomes largely depend on appropriate lens sizing, as it directly relates to the post-operative vault.

Additional risk factors, such as high myopia, can concurrently increase surgical risk. Particularly, cataract surgery in young patients with high myopia (greater than −5.0 D) has been linked to increased post-operative complications compared to emmetropic eyes [16,17,18]. Given that over two million implantable collamer lenses (ICLs) have been implanted worldwide and that a percentage of these patients will develop early cataracts (a late complication of pIOL) and almost all will eventually develop age-related cataracts, it is crucial to understand the visual prognosis for these typically highly myopic patients after cataract surgery [19].

This study is a retrospective case series on a large group of patients who underwent ICL surgery and later developed visually significant cataracts. The primary outcome focuses on visual prognosis after cataract removal, and we also discuss the correlation between vault height and cataract formation as an important risk factor. Although several case series have been reflected in the literature originating from Europe and Asia, this is the first large cohort in the United States.

## 2. Materials and Methods

### 2.1. Study Design

This study is a retrospective case series analysis of protected medical records from patients who developed visually significant cataracts after original ICL implantation. All data were obtained from a single refractive surgery center (Hoopes Vision) and patient information was de-identified. After performing an extensive chart review, patients were included if they developed a cataract after ICL implantation and underwent concurrent explantation with corrective cataract surgery. Exclusion criteria included those with a history of intraocular surgery before ICL implantation. The consent and study procedures were approved by Hoopes Vision Ethics Committee and adhered to the tenets of the Declaration of Helsinki. The Biomedical Research Alliance of New York (BRANY) Institutional Review Board (IRB) approved this retrospective study (#A20-12-547-823).

### 2.2. Study Measurements

Initially patients’ ICL sizing was determined using a combination of the average biometric measurements from Pentacam HR (Oculus, Arlington, WA, USA), G4 Galilei (Ziemer, Switzerland), NIDEK OPD-Scan III system (Gamagori, Japan), Zeiss IOLMaster 700 version 1.90.12.05 (Carl Zeiss Meditec AG, Jana, Germany), Lenstar LS 900 version i9.6.3.0 (Haag-Streit, Koeniz, Switzerland) and Presurgical VuMax ultrasound bio-microscopy (UBM) (Sonomed Escalon, New Hyde Park, NY, USA). The selection of the proper Visian V4 ICL size before 2015 was based on the Rivera, FDA, and Optimized FDA nomograms utilizing measurements of white-to-white (WTW) and anterior chamber depth (ACD). After 2015, the selection was based on Parkhurst nomogram utilizing measurements of aqueous depth, sulcus-to-sulcus, and crystalline lens rise (CLR). CLR was defined as the distance between the anterior pole of the crystalline lens and the sulcus-to-sulcus plane. The lenses were subsequently ordered through the STAAR Surgical Online Calculating and Ordering System (OCOS, Monrovia, CA, USA https://evo-ocos.staarag.ch/Live/; accessed on 15 April 2024).

Concerning cataract surgery, the intraocular lens (IOL) model and power determination based on the target refraction were selected utilizing biometric measurements obtained from the IOLMaster based on the speed alternative: “phakic”. The following measurements were collected: WTW, ACD, flat keratometry (K1), steep keratometry (K2), central cornea thickness (CCT), lens thickness (LT), and axial length (AL). The IOL was then calculated using either the SRK/T or the Barrett Universal II formulas.

With regards to CE, patients were evaluated post-operatively at one week, one month, and six months after CE. Outcomes such as uncorrected distance visual acuity (UDVA), corrected distance visual acuity (CDVA), spherical equivalent (SEQ), and refractive cylinder were measured and assessed. Additionally, the objective vault measurements were collected at two time periods: one week following ICL implantation and one week before cataract removal.

### 2.3. Surgical Techniques

A 2.4 mm temporal clear corneal incision was created, followed by the injection of a dispersive viscoelastic agent to protect the endothelium and maintain the anterior chamber. A cohesive viscoelastic agent was injected between the ICL and the crystalline lens. An O’Gawa instrument was used to carefully lift the proximal end of the ICL away from the crystalline lens to avoid inadvertent damage to the capsular integrity. The two proximal foot plates were brought over the iris near the corneal incision. The ICL was then grasped with 0.12 mm-toothed forceps, folded upon itself, and carefully removed from the anterior chamber through the corneal incision. An additional dispersive viscoelastic agent was injected into the anterior chamber. A 5.5 mm continuous curvilinear capsular-hexis (CCC) was created. Hydro-dissection and hydro-delineation were performed using an irrigation cannula. The cataract and cortical lens material were removed using phacoemulsification and irrigation and aspiration handpieces, followed by thorough capsular polishing using a bimanual technique and a curved 27-gauge Jensen capsule polisher cannula (Ambler Surgical, Exton, PA, USA). A cohesive viscoelastic agent was then injected into the capsular bag. An appropriate IOL was selected and placed inside the capsular bag, ensuring complete coverage of the optic edge with the CCC. The residual viscoelastic agent was removed, and all wounds were confirmed to be self-healing.

The post-operative pharmacologic regimen included moxifloxacin 0.5% ophthalmic solution four times daily for one week, prednisolone acetate 1% ophthalmic suspension drops four times daily with a taper over one month, and ketorolac 0.5% ophthalmic solution twice daily for six weeks.

### 2.4. Statistical Methods

The data were analyzed using Statistical Package for the Social Studies (SPSS) version 29 (IBM Corp, New York, NY, USA). The mean values, standard deviations, and student *t*-tests were calculated for parameters with a normal distribution. Normality was evaluated through a Shapiro–Wilk test. Data found to be nonparametric were evaluated and analyzed utilizing median, interquartile ranges (IQR), and the Wilcoxon/Mann–Whitney test depending on paired vs unpaired, to test for statistical significance. The nine standardized graphs were constructed utilizing mEYEstro software (MathWorks Inc, Natick, MA, USA) [20]. A regression model was used to assess the relationship between vaults (predictor variable) and years until CE (outcome variable). A square root transformation was applied to the outcome variable.

## 3. Results

### 3.1. Demographics

Between May 2008 and March 2022, a total of 772 patients underwent ICL implantation at our surgical site. Among this cohort, 32 patients (51 eyes) developed visually significant cataracts and subsequently underwent cataract extraction between August 2010 to January 2024, resulting in a cumulative incidence of 4.1% over 14 years. Additionally, four patients (seven eyes) underwent ICL implantation at other surgical centers but sought follow-up treatment at Hoopes, resulting in a total of 36 patients (58 eyes) managed via cataract extraction. The mean age of our study group at the time of ICL implantation was 41 ± 7 years (range: 26 to 55 years) (Table 1), on average six years older than the mean age of the entire ICL cohort (35 ± 8 years, range: 18 to 66 years).

**Figure 1:**
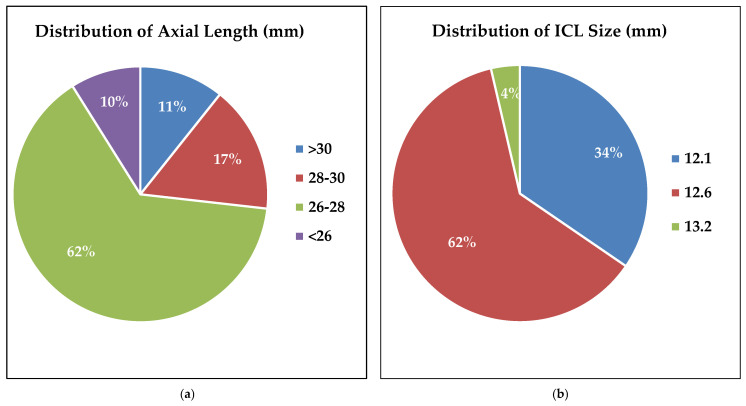
(**a**) Illustrates the distribution of axial length by percentage of patients within the following range: >30 mm = 11%, 28–30 = 17%, 26–28 = 62%, and <26 = 10%. (N = 58). (**b**) Demonstrates the distribution of Visian ICL V4 pIOL sizes. Three sizes of ICL were represented in our patient population. 12.1 mm = 34%, 12.6 mm = 62%, and 13.2 mm = 4%. (N = 55).

**Figure A1:**
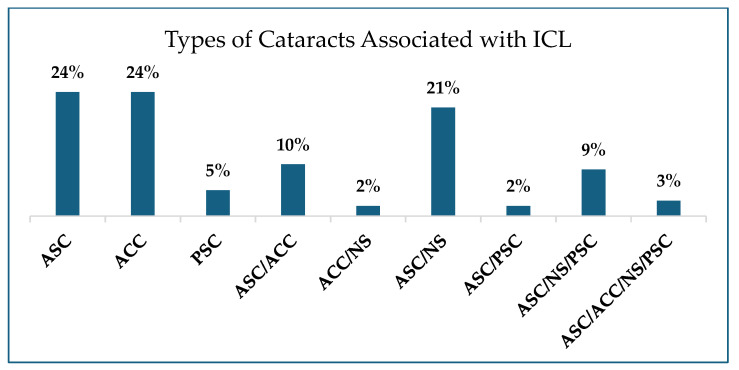
Types of Cataracts Associated with ICL. Presented by a percentage of patients with either a combination of cataract types or solely one type. ASC = anterior subcapsular cataract, ACC = anterior cortical cataract, PSC = posterior subcapsular cataract, NS = nuclear sclerosis. ICL = implantable collamer lens. (N = 58).

Our patient population consisted of 15 females and 21 males with a mean age at the time of cataract extraction of 47 ± 8 years, resulting in an average time from ICL implantation to CE of 6 ± 4 years. Pre-operative biometric findings of the patients who underwent cataract extraction are included in Table 1. The mean axial length was 27.6 ± 1.7 mm (range: 23.8 to 31.8 mm) (Figure 1). Various types of cataracts were observed. The most common was anterior subcapsular cataract (ASC), which appeared either in isolation or in combination with other types of cataracts in 69% of patients. This was followed by anterior cortical cataract (ACC), observed either alone or combined with other types of cataracts in 39% of patients (Appendix A, Figure A1).

Six patients (seven eyes) required additional procedures between ICL implantation and cataract removal. Two eyes underwent post-operative enhancement with photorefractive keratectomy (PRK) at six and ten months, respectively. Additionally, four patients (five eyes) underwent limbal relaxing incisions (LRI) for correction of astigmatism. More importantly, two patients (two eyes) experienced late rhegmatogenous retinal detachments (RDs) requiring therapeutic vitrectomies at three- and seven-years post-ICL implantation. After vitrectomy, cataract surgery was indicated after three months and 1.7 years, respectively.

The Visian V4 ICL model was implanted in all eyes. Patients received one of three of the following ICL sizes (mm): 12.1 (34%), 12.6 (62%), and 13.2 (4%) (Figure 1b). Concerning cataract surgery, 17 types of posterior chamber intraocular lens models and lens types were utilized (Table A1). The distribution of ICL and IOL powers used can be found in Table 1.

### 3.2. Visual Outcomes

**Figure 2:**
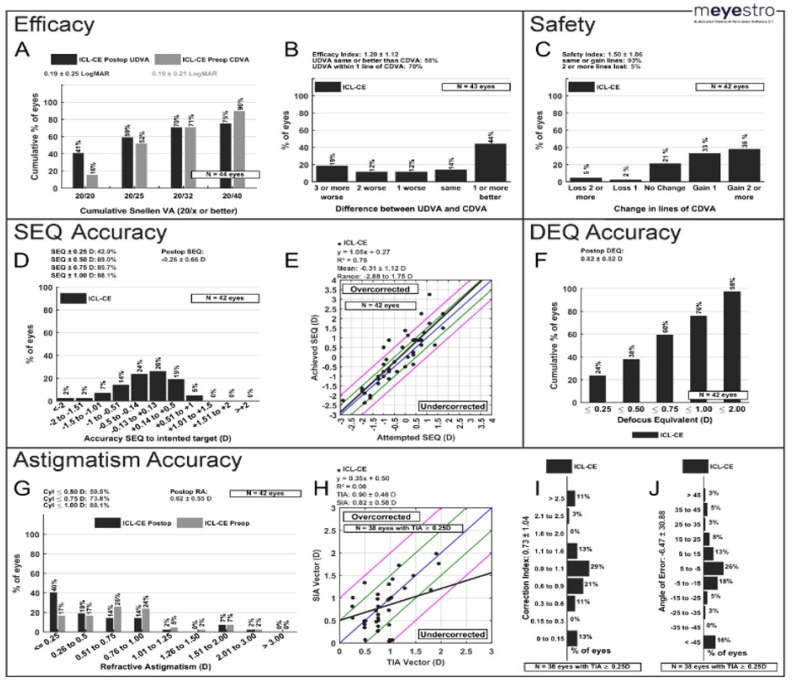
Visual Outcomes six months after CE (n = 44) (**A**) Post-operative uncorrected distance visual acuity (UDVA) versus pre-operative corrected distance visual acuity (CDVA). (**B**) Efficacy: change in Snellen lines from pre-operative CDVA to post-operative UDVA. (**C**) Safety: change in Snellen lines from pre-operative CDVA to post-operative CDVA. (**D**) Spherical Equivalent (SEQ) Accuracy: accuracy of post-operative spherical equivalent refraction to target. (**E**) Attempted versus achieved spherical equivalent refraction, with linear regression and correlation values; the black line represents the equation y = x, and the closer the regression line is to the black line, the more accurate the results. (**F**) Defocus Equivalent (DEQ) Accuracy (**G**) Change in refractive astigmatism. (**H**) TIA vs. SIA; Blue Line: target; Between green lines: within ±0.50 D of target; Between pink lines: within ±1.0 D of target. (**I**) Histogram of correlation Index; (**J**) Angle of Error. CE = Cataract Extraction, TIA = Target-induced astigmatism, SIA = surgically induced astigmatism, ICL = Implantable collamer lens, D = Diopter.

Out of the 58 eyes, measurements for visual outcomes after CE were available for 58, 50 and 44 eyes at one week, one month and six months, respectively. Pre-operatively, CDVA was 20/40 or better in 90% of eyes, 20/32 or better in 71% of eyes, 20/25 in 52% of eyes, and 20/20 or better in 16% of eyes (Figure 2A). Efficacy index after cataract extraction at one week, one month, and six months was 1.31 ± 1.06, 1.41 ± 1.19, and 1.20 ± 1.11, respectively (*p* > 0.05). At six months, 41% of eyes achieved a cumulative UDVA of 20/20 or better, compared to only 16% pre-operatively with a cumulative CDVA of 20/20 or better (Figure 2A). Additionally, 70% of eyes had a post-operative UDVA within one line of pre-operative CDVA (Figure 2B).

Safety index after cataract extraction at one week, one month, and six months was 1.63 ± 1.08, 1.71 ± 1.1, and 1.50 ± 1.06, respectively. At six months, 71% of eyes gained one or more lines of CDVA, 21% of eyes had no change in lines, and 7% of eyes lost one or more lines of CDVA (Figure 2C). This 7% was attributed to the following: irregular astigmatism, ocular surface dryness and mild posterior capsular changes.

### 3.3. Refractive Outcomes

Mean SEQ after cataract extraction at one week, one month, and six months was 0.08 ± 0.9 D, 0.063 ± 0.9 D, 0.16 ± 1.0 D, respectively, with no significant differences between time points (*p* > 0.05) (Table 2). Concerning predictability, 43% and 69% of eyes were within ±0.25 D and ±0.50 D of SEQ target at six months, respectively (Figure 2D). Attempted vs. achieved SEQ regression analysis also showed a mean predictability of −0.31 ± 1.12 D and a mean regression line value of 1.05x + 0.27 (Figure 2E). At six months, the mean defocus equivalent (DEQ) was 0.82 ± 0.52 D, with 38% of eyes achieving a DEQ of ≤0.50 D (Figure 2F).

Concerning astigmatism after cataract extraction, 40% and 60% of eyes had a refractive cylinder of ≤0.25 D and ≤0.50 D, respectively, at six months, compared to 17% and 34% of eyes, respectively, during the pre-operative period (Figure 2G). Mean refractive cylinder of all eyes at six months was 0.62 ± 0.55 D. 88.4%, 74.4%, and 58.1% of patients at six months had a refractive cylinder ≤1.0, 0.75, and 0.50, respectively. The mean target-induced astigmatism (TIA) vector was 0.90 ± 0.48 D, whereas the mean surgically induced astigmatism (SIA) vector was 0.82 ± 0.58 D (Figure 2H). At six months, the correction index was 0.73 ± 1.04 (Figure 2I) and the mean angle of error was –6.47 ± 30.88 (Figure 2J).

### 3.4. Vault Analysis

**Figure 3:**
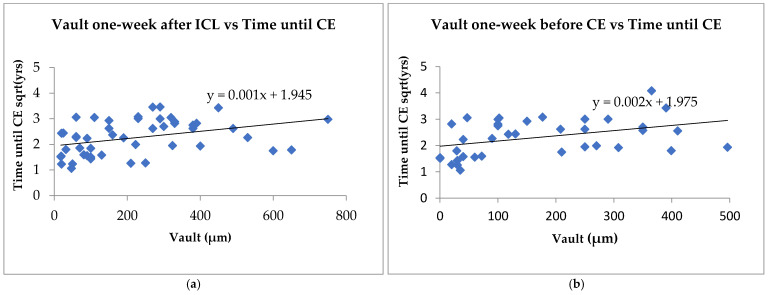
Regression model of the relationship between vault height and time from ICL implantation to cataract extraction (CE). (**a**) Demonstrates the vault one week after implantable collamer lens (ICL) vs square root of the time until ICL. (n = 48). (**b**) Regression model of the relationship between late vault and the square root of the time until CE. R^2^ values = 0.16 (**a**) and 0.14 (**b**) (n = 36).

The median vault height one week after ICL implantation and one week before CE was available for 48 and 36 eyes, respectively. The median early vault was 190 µm (IQR: 75.5 to 327.00) and the median late vault was 110 µm (IQR: 40.00 to 496.00). The vaults significantly decreased in size during the time between ICL implantation and cataract extraction (*p =* 0.01). Figure 3a models the relationship between vault at one week after ICL and the square root of the time until CE with estimated parameters for β0 and β1 of 1.945 (*p =* 1.26 × 10^−17^) and 0.001 (*p =* 0.008), respectively. Figure 3b models the relationship between vault one week before CE and the square root of the time until CE with estimated parameters for β0 and β1 of 1.975 (*p =* 1.48 × 10^−13^) and 0.002 (*p =* 0.015), respectively. Both models demonstrate a significant positive association between vault height and time until CE (R^2^ values = 0.16 and 0.14, respectively).

## 4. Discussion

Advancements in research and technology have significantly improved the outcomes of ICL surgery. These improvements include enhanced diagnostic capabilities for ICL sizing estimation and refined nomograms that offer better predictability of post-operative vaulting [21]. Modern ICL lens models, such as EVO/EVO+ and EVO viva, feature larger optical zones, a 360 µm central port, and presbyopia correction for patients up to 55 years old [22]. Consequently, surgeons are now able to offer ICL surgery to a broader range of patients in terms of age and myopia [22,23,24,25].

Despite these advancements, cataract formation remains a significant safety concern in phakic posterior chamber intraocular lens surgery, particularly before the advent of the central aqua-port feature. The aqua-port, now standard in most myopic PC pIOL models, enhances aqueous fluid flow around the lens, reducing the need for peripheral iridotomies and subsequently lowering the risk of cataracts. Historically, anterior subcapsular cataract incidences were about 6.1%, with a 1.2% chance of becoming visually significant. Recent studies, including a review by Montes-Mico et al., have shown that the prevalence of cataracts following V4c ICL with the central port has reduced to 0.17%. Factors contributing to this improvement include the experience of the surgeon and refined surgical techniques [26,27]. Clinicians should remain vigilant about patient-specific risks that may lead to earlier-than-expected cataract formation. When cataract surgery becomes necessary, it is crucial to provide patients with a clear prognosis of their visual outcomes.

There was a significant improvement in visual acuity after cataract extraction, like the improvement seen by Vargas et al., who reported an average post-operative UDVA and CDVA of 20/35 and 20/25, respectively [26,27,28,29,30]. Our efficacy index of 1.2 at six months reflects that cataracts were visually significant. Similarly, Vargas et al. and Kamiya et al. reported good efficacy after cataract removal with an index of 0.8 and 1.13, respectively [28,30]. Kamiya et al. found a good safety index of 2.51 ± 3.35 at 3 months post-operatively with a significant improvement in best-corrected visual acuity after cataract removal [30]. This supports our safety index of 1.50 at six months post-operatively. It is important to note that, although 71% of eyes gained Snellen lines, 7% of eyes lost lines of Snellen CDVA. As mentioned in our Results section, this finding was attributed to irregular astigmatism, with one eye experiencing accidental trauma after cataract surgery, ocular surface dryness (the etiology of which was not described in the patient’s chart), and posterior capsular changes. Our predictability at six months was good, with 86% and 69% of eyes within ±1.0 D and ±0.50 D, respectively, of the calculated target. Similarly, Morales et al. reported a predictability of 71.4% of eyes within ±1.0 D. SEQ after cataract surgery was stable at one week, one month, and six months without any significant differences between time points [31]. In addition, the IOL of choice addressed refractive astigmatism after cataract removal, as seen in Figure 2G–J. Vargas et al. found that 28.5% of eyes had a refractive cylinder post-operative value of ≤0.50 D [28], while 59% of eyes in our population exhibited a value of ≤0.50 D. There were no significant differences in post-operative astigmatism between ICL implantation and cataract extraction.

The median vault height one week after ICL was 190 µm, which decreased to 110 µm at one week before CE, suggesting a significant decrease in vault height over time. This is consistent with findings in other studies, such as that of Ouchi et al., who showed a gradual decrease in vault height over time in patients with both horizontal and vertical fixation [32]. However, 42% of eyes in our study fell below 200 µm after initial implantation, well below the acceptable vault height [5]. One may argue that this group was at a high risk of developing cataracts to begin with, which became evident given time. Additionally, as shown in Figure 3, there was a significant positive linear relationship between the time from ICL implantation to cataract extraction in both the vault measurements. This supports previous reports attributing low vault height as the primary contributor to ICL-induced cataracts due to contact between the lens and the ICL implant [14]. Our study group developed visually significant cataracts in less than a decade after ICL, further strengthening the relationship between small vault height and faster cataract development. As a result, we believe that our study group consisted of undersized ICL implants. One of the most common contributing factors to small height is inappropriate ICL sizing [3,25]. Interestingly, 34% of our eyes had a 12.1 mm lens and 62% had a 12.6 mm lens (Figure 1b). In other studies, with similar demographics, most lenses were either 12.6 mm or 13.2 mm sizes with minimal 12.1 mm lenses [33,34,35]. Currently, there are only four models available (12.1 mm, 12.6 mm, 13.2 mm, and 13.7 mm). Taking this into consideration, perhaps if some of our patients had been equipped with a larger lens, then this could have delayed or mitigated cataract complications. Moreover, our patient population may have benefited from additional ICL models in intermediate sizes.

Conversely, additional inherent risk factors within our study population potentially contributed to ICL-induced cataract formation. These include age >40–45, myopia stronger than −12.0 D, and pre-existing lens opacity [5]. Our study group exhibited several of these factors including two eyes (out of 58 eyes) documented pre-existing lens opacities, 28% of eyes (17 eyes) with a SEQ (D) >−12, and 53% of patients (31 patients) being older than 40 years at the time of implantation. This study group undergoing ICL implantation was six years older than the average age of the entire ICL cohort, possibly compounding their risks of cataract formation. Since patients who undergo ICL tend to have a higher myopic power, it is important to take into consideration the innate risks associated with myopia, such as developing cataracts at an earlier age [36]. A retrospective study by Jeon et al. found the mean age at the time of cataract surgery was 60 ± 12 years in the group with AL > 26 mm, in contrast with the control group (AL < 26 mm) with a mean age of 67 ± 11 years [37]. Our study group revealed an even more profound difference compared with Jeon et al.’s control group, with a mean age of 47 ±8 years at the time of cataract removal within a population where 90% of patients had an AL > 26 mm. Another significant risk factor within our study group was that of patients who developed RDs three to seven years after ICL implantation, managed with immediate therapeutic vitrectomies. Consequently, this led to cataract formation three months and 1.7 years post-vitrectomy, respectively.

Limitations of this study are related to the inherent nature of a retrospective study. One of these limitations, which deprived us of critical information considering that pIOLs, including ICLs, have been associated in some studies with progressive endothelial cell loss [38,39,40], was that we did not have specular microscopy data. This information would have provided a clearer understanding of the safety of the Visian ICL V4 and the potential for corneal complications after late secondary phacoemulsification in these eyes. Another limitation is that we used data from both eyes of individuals, instead of randomly selecting one eye from each patient. We realize the existence of bias from between-eye correlation; however, the sample size was increased in efforts to increase the statistical power of the study. Some may also question why cataract surgery was performed when the mean CDVA prior to cataract extraction was 0.097 (Table 2). However, many of our patients complained of glare, nighttime driving issues, and cloudy vision, symptoms attributed to clinically observed cataracts. Furthermore, 69% of our patients had ASC cataracts, which may lead to better correction of Snellen visual acuity but can also cause glare symptoms, as noted by many of our patients. Some may also argue that our 69% predictability of SEQ within 0.50 D of the target is not reflective of the refractive predictability of modern phacoemulsification. However, it is important to note that the eyes in this study were severely myopic, with a high mean axial length (27.6 ± 1.7 mm), which could have potentially influenced our results of predictability at 6 months.

Concerning the incidence of visually significant cataract formation after ICL implantation, the literature reports a broad incidence range 0.3–28% [5]. Our estimated incidence of 4.1% over 14 years was on the lower end, whereas Choi et al. report an incidence of 12.1% over 10 years [41]. We recognize that it is common for patients to be lost to follow-up, as was the case with some of the patients in our study, therefore we believe this underestimates the risk of developing visually significant cataract. However, we believe our incidence is more reflective of Caucasians within the United States, which has not been reported before. Some may also argue that utilizing safety and efficacy indices to assess visual outcomes after cataract surgery may be inappropriate, as these results could be artificially inflated due to the poor visual acuity prior to surgery and the significant improvement in vision afterward. However, considering that other studies [28,30] in the current literature have evaluated safety and efficacy indices after cataract surgery in patients with a history of ICL implantation, we found it necessary to include and compare our results with those of the mentioned studies. Similar to our study, most of the aforementioned studies evaluating cataract surgery outcomes after ICL have limited follow-up (up to one year), and time will tell if these improvements result in favorable long-term outcomes. Furthermore, a larger cohort is necessary for investigating whether IOL model/specification impacts visual outcomes, an important aspect that warrants further study.

## 5. Conclusions

In conclusion, this study supports good visual prognosis in patients who have previously received ICL implants, then undergo cataract extraction. Furthermore, this study, supported by previously published literature, demonstrates excellent visual outcomes in patients who require cataract extraction after ICL implantation [17,18,19,20,21,22,23,24,25,28,29,30,31]. However, low vaults remain a major prognostic factor in developing cataracts sooner than chronologically expected, particularly in those with inherently higher risk, such as those with high pre-operative myopia and of older age. Technological advancements continue to expand the selection criteria for those who can safely undergo ICL surgery by improving proper ICL sizing and vault predictability. As the approval for ICL continues to rise, understanding patient-specific risks can offer an informed discussion concerning the post-operative expectation and prognosis after cataract surgery in patients with prior history of ICL implantation.

## Figures and Tables

**Table 1:** Demographics and Pre-operative CE Biometric Values.

Variable	Mean (±SD)	Range
Sex assigned at birth	43% (Female)	57% (Male)
Age at time of surgery (y)	41 (±7)	26 to 55
Age at time of cataract extraction (y)	47 (±8)	30 to 71
Time from ICL implantation to CE (y)	6 (±4)	1 to 18
Keratometry K1 (D)	43.9 (±1.75)	39.8 to 48.3
Keratometry K2 (D)	45.2 (±1.99)	40.2 to 49.9
White-to-white (mm)	12.14 (±0.43)	11.4 to 13
Anterior chamber depth (mm)	3.3 (±0.4)	2.7 to 4.2
Corneal thickness (μm)	544 (±40.02)	446 to 628
Axial Length (mm)	27.6 (±1.7)	23.8 to 31.8
Lens thickness (mm)	4.4 (±0.4)	3.3 to 4.95
Visian ICL V4 Power (D)	−11.6 (±2.3)	−16 to −7
IOL Power (D)	8.3 (±3.6)	−4 to 16.5

D = diopter, SD = Standard Deviation.

**Table 2:** Mean Spherical Equivalent, Cylinder and Visual Acuity.

Parameter	Before ICL	After ICL (6 mo)	Before CE	After CE (6 mo)
Spherical Equivalent (D)	−10.9 (±2.75)	0.12 (±0.72)	−0.08 (±1.37)	0.16 (±1.05)
Cylinder (D)	−1.38 (±0.98)	−0.68 (±0.65)	−0.08 (±0.89)	−0.59 (±0.58)
LogMAR UDVA	1.78 (±0.23)	0.11 (±0.16)	0.477 (0.243) *	0.21 (±0.27)
LogMAR CDVA	0.00 (0.30) *	0.00 (0.00) *	0.097 (0.204) *	0.06 (±0.22)

D = diopter, (*) denotes interquartile range, ICL= Implantable collamer lens, CE = cataract extraction. Our predetermined α was 0.05. (After ICL (6 mo), n = 38).

## Data Availability

The data presented in this study are available on request from the corresponding author due to ethical restrictions.

## Appendix A

**Table A1:** Intraocular Lens (IOL) Model and Lens Type.

Company	Model	Lens Type
Johnson and Johnson (New Brunswick, NJ, USA)	AR40E, DIB00, DXR00V, ZCB00, ZMA00	hydrophobic acrylic Tecnis
	DFW150, DIU150, DIU525, DXW225	hydrophobic acrylic Tecnis Toric
	ZKB00, ZMB00	hydrophobic acrylic Tecnis multifocal
Alcon (Geneva, Switzer-land)	SN6AT4	hydrophobic acrylic 1-piece Toric
	MA60MA	hydrophobic acrylic 3-piece
Bausch + Lomb (Laval, Canada)	AT52SE	Silicone Elastomer (Biosil)
	MX60E	hydrophobic acrylic 1-piece
STAAR (Monrovia, CA, USA)	AQ5010	Silicone 3-piece
	CC4204A	Collagen-copolymer
